# Static Stretch Training versus Foam Rolling Training Effects on Range of Motion: A Systematic Review and Meta-Analysis

**DOI:** 10.1007/s40279-024-02041-0

**Published:** 2024-05-17

**Authors:** Andreas Konrad, Shahab Alizadeh, Saman Hadjizadeh Anvar, Josef Fischer, Josefina Manieu, David G. Behm

**Affiliations:** 1 Institute of Human Movement Science, Sport and Health, Graz University, Mozartgasse 14, 8010 Graz, Austria; 2 School of Human Kinetics and Recreation, Memorial University of Newfoundland, St. John’s, NL Canada; 3 Human Performance Lab, Department of Kinesiology, University of Calgary, Calgary, AB Canada

## Abstract

**Background:**

Long-term static stretching as well as foam rolling training can increase a joint’s range of motion (ROM). However, to date, it is not clear which method is the most effective for increasing ROM.

**Objective:**

The purpose of this systematic review and meta-analysis was to compare the effects of static stretching and foam rolling training on ROM.

**Methods:**

The literature search was performed in PubMed, Scopus, and Web of Science to find the eligible studies. Eighty-five studies (72 on static stretching; and 13 on foam rolling) were found to be eligible with 204 effect sizes (ESs). For the main analyses, a random-effect meta-analysis was applied. To assess the difference between static stretching and foam rolling, subgroup analyses with a mixed-effect model were applied. Moderating variables were sex, total intervention duration, and weeks of intervention.

**Results:**

Static stretch (ES =  − 1.006; *p* < 0.001), as well as foam rolling training (ES =  − 0.729; *p* = 0.001), can increase joint ROM with a moderate magnitude compared with a control condition. However, we did not detect a significant difference between the two conditions in the subgroup analysis (*p* = 0.228). When the intervention duration was ≤ 4 weeks, however, a significant change in ROM was shown following static stretching (ES =  − 1.436; *p* < 0.001), but not following foam rolling (ES =  − 0.229; *p* = 0.248). Thus, a subgroup analysis indicated a significant favorable effect with static stretching for increasing ROM compared with foam rolling (*p* < 0.001) over a shorter term (≤ 4 weeks). Other moderator analyses showed no significant difference between static stretch and foam rolling training on ROM.

**Conclusions:**

According to the results, both static stretching and foam rolling training can be similarly recommended to increase joint ROM, unless the training is scheduled for ≤ 4 weeks, in which case static stretching demonstrates a significant advantage. More studies are needed with a high-volume foam rolling training approach as well as foam rolling training in exclusively female participants.

## Key Points

**Table:** 

Static stretching as well as foam rolling training can increase the range of motion of a joint in the long term with a moderate magnitude of change.
A subgroup analysis showed no significant difference between static stretching and foam rolling training for the increase in range of motion.
When the intervention duration was ≤ 4 weeks, a significant favorable effect with static stretching for an increasing range of motion compared with foam rolling was shown.

## Introduction

Static stretch (SS) training is the most commonly used technique for long-term increases in the range of motion (ROM) [1–5]. Commonly, with SS, the joint is held at the maximum ROM at a specific stretch intensity (e.g., until the point of discomfort) [6]. Whilst SS training is similarly effective for increasing ROM as proprioceptive neuromuscular facilitation training, it can induce greater ROM increases compared with dynamic stretch training [7].

In addition to stretch training, other strategies such as resistance training, when performed through the whole ROM [8], or foam rolling [9] can also increase joint ROM long term. According to previous meta-analyses, long-term stretch training and resistance training can be considered similarly effective for increasing the ROM (effect size [ES] = 0.08; *p* = 0.79) [8, 10].

However, there is a lack of evidence available comparing the effectiveness between long-term foam rolling and stretch training for increasing the ROM. As the two techniques are very frequently used in sports practice or therapy, it is very important to discover whether there is a difference in the long-term increase in ROM between SS and foam rolling. Konrad et al. [11] in their meta-analysis presented only three studies that explored both long-term stretch training as well as foam rolling training. Stretch training demonstrated a non-significant small-magnitude (ES = 0.516; *p* = 0.12) advantage over foam rolling training for increasing ROM. Again, it must be emphasized that only three studies were included in this analysis and hence, no real conclusion can be drawn if stretching might be more efficient compared to foam rolling for the increase in ROM. When considering SS effects in isolation, a recent meta-analysis reported an increase in ROM following a long-term SS program with a moderate magnitude of change compared with a control condition (ES = 1.005) [7]. Similarly, following long-term foam rolling interventions, a moderate-magnitude increase in ROM was reported in another meta-analysis by Konrad et al. [11] (ES = 0.823). Although there is a similar magnitude of change between SS and foam rolling, the ES in static stretching was ~ 22% higher and hence, it is not unlikely that SS might be more efficient for long-term ROM increases compared to foam rolling. However, to date, no meta-analysis has compared all the available evidence of the ESs between isolated SS studies to isolated foam rolling studies.

Therefore, this systematic review and meta-analysis aims to examine the potential differences in the ES between SS and foam rolling on joint ROM in healthy participants. Moreover, potential moderating variables such as sex, total intervention duration, and weeks of intervention will be further considered.

## Methods

This review was conducted according to the PRISMA (Preferred Reporting Items for Systematic Reviews and Meta-Analyses) guidelines and the suggestions from Moher et al. [12] for systematic reviews with a meta-analysis.

### Search Strategy

Previously, our research group published meta-analyses on the long-term effects of stretching [7] and foam rolling [9] on ROM. Consequently, to identify all the relevant studies, a search for additional papers published after the search from the aforementioned studies until 4 July, 2023 was conducted. The electronic literature search for the current review was performed in PubMed, Scopus, and Web of Science using the same search codes as were used for the previous meta-analyses [7, 9]. Using AND and OR Boolean operators, a systematic search was conducted using the following keywords for finding all stretching studies: flexibility, “range of motion”, extensibility, stretch*. In addition to the aforementioned keywords, the studies were filtered using the subsequent keywords to include controlled trials: “randomized controlled trial,” “controlled clinical trial,” “randomized,” “placebo,” “randomly,” and “trial”. Furthermore, to exclude animal studies, a NOT operator with the following MeSH Term “exp animals/not humans” was added. For example, the following search query was used in PubMed: ((((“flexibility”[Title/Abstract]) OR (“range of motion”[Title/Abstract])) OR (“extensibility”[Title/Abstract])) AND (“stretch*”[Title/Abstract])) AND ((((((((“randomized controlled trial”[Publication Type]) OR (“controlled clinical trial”[Publication Type])) OR (“randomized”[Title/Abstract])) OR (“placebo”[Title/Abstract])) OR (“clinical trials as topic”[MeSH Terms])) OR (“randomly”[Title/Abstract])) OR (“trial”[Title/Abstract])) NOT (exp animals/not humans[MeSH Terms])). Additionally, to find eligible studies on foam rolling, the following search code was used in all databases: (“chronic effects” OR “training effects” OR effects OR “long-term” OR intervention) AND (“foam rolling” OR “self-myofascial release” OR “roller massage” OR “foam roller”) AND (flexibility OR “range of motion”). The updated systematic search was conducted by two independent researchers (JF, JM). Initially, the articles were screened by their title and then abstract. If the content remained unclear, the full text was retrieved for further screening and identifying the relevant papers. Following this independent screening process, the researchers compared their findings. Disagreements were resolved by jointly reassessing the studies against the eligibility criteria.

### Inclusion and Exclusion Criteria

This review considered studies that investigated the training effects of stretching and foam rolling on joint ROM in healthy participants. The studies were included when they were either randomized controlled trials or controlled trials with an intervention duration ≥ 2 weeks [13]. This implied that studies that were dealing with the short-term effects of stretching (or interventions shorter than < 2 weeks), investigated any combined treatment (e.g., stretching combined with strength training), or had another treatment as a control condition were excluded. Moreover, review papers, case reports, special communications, letters to the editor, invited commentaries, conference papers, or theses were excluded.

### Extraction of the Data

From the included papers, the characteristics of the participants (i.e., age, sex), sample size, characteristics of the intervention (i.e., total intervention duration in seconds, weeks of intervention), and results of the main variables (flexibility parameters) were extracted. For the flexibility parameters, pre-intervention and post-intervention values plus standard deviations of the foam rolling and control groups were extracted. If some of the required data were missing in the included studies, the authors of the studies were contacted via e-mail or similar channels (e.g., Research Gate).

### Statistics and Data Synthesis

The meta-analysis was performed using Comprehensive Meta-Analysis software, according to the recommendations of Borenstein et al. [14]. By applying a random-effect meta-analysis, the ES in terms of the standardized mean difference was assessed. If any study reported more than one ES, the mean of all the outcomes (ESs) within one study was used for the analysis and was defined as combined (as suggested by Borenstein et al. [14]). To determine differences between the ESs of static stretching and foam rolling training on ROM, subgroup analyses were performed. A mixed-effect model was used for this purpose and *Q*-statistics were applied [14]. Although there is no general rule of thumb [14], we only performed subgroup analyses when there were three or more studies included in the respective subgroups. Consequently, it was possible to perform subgroup analyses with the following moderators: male participants, total intervention duration < 3600 s, > 4 weeks intervention duration, and ≤ 4 weeks intervention duration. It was not possible to perform such a subgroup analysis with female participants or with a total intervention duration ≥ 3600 s because only two foam rolling studies included these moderators. An analysis on age was not performed as almost all studies on foam rolling were dealing with participants less or equal to 25 years of age. A cut-off of 4 weeks was chosen because of a previous analysis on foam rolling [11]. Additionally, the 3600-s cut-off was chosen according to another recent stretching review [15] in which 10 weeks training with three sessions per week (2 × 30-s stretches) were assumed. This cut-off represents a typical stretch protocol in sports practice [15, 16].

According to the recommendations of Hopkins et al. [17], the effects for a standardized mean difference of < 0.2, 0.2–0.6, 0.6–1.2, 1.2–2.0, 2.0–4.0, and > 4.0 were defined as trivial, small, moderate, large, very large, and extremely large, respectively. *I*^2^ statistics were calculated to assess the heterogeneity among the included studies, and thresholds of 25%, 50%, and 75% were defined as having a low, moderate, and high level of heterogeneity, respectively [18, 19]. An alpha level of 0.05 was defined for the statistical significance of all the tests.

### Risk of Bias Assessment and Methodological Quality

The methodological quality of the included studies was assessed using the PEDro scale for the additional papers not found in the previous meta-analyses [7, 9]. In total, 11 methodological criteria were rated by two independent researchers (SA, SHA) and were assigned either one or no points. Hence, higher scores indicated better methodological quality of the study. In cases of conflict between the researchers, the methodological criteria were reassessed and discussed. Moreover, statistics of the Egger’s regression intercept test and visual inspection of the funnel plot were applied to detect possible publication bias.

## Results

### Results of the Search

**Fig. 1:**
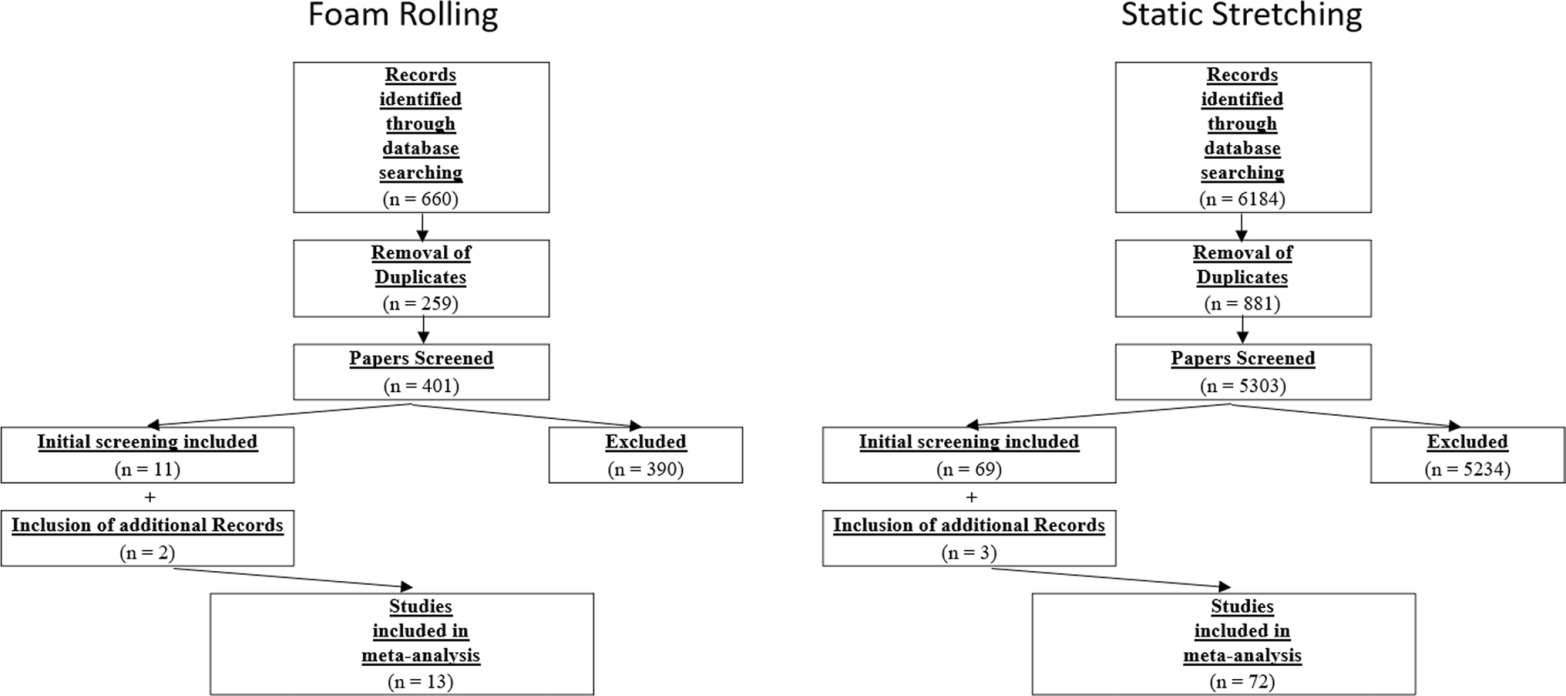
PRISMA (Preferred Reporting Items for Systematic Reviews and Meta-Analyses) identification and selection flowchart

Overall, after removal of the duplicates, 5704 papers were screened, from which 80 papers were found to be eligible for this review. However, following the additional search of the references (search through the reference list) and citations (search through Google Scholar) of the 80 already included papers, five more papers were identified as relevant. Therefore, in total, 85 papers were included in this systematic review and meta-analysis. Figure 1 shows the search process for both static stretching and foam rolling.

Overall, 156 ESs could be extracted from 72 eligible studies for static stretching as well as 48 ESs from 13 eligible studies for foam rolling. Table 1 presents the characteristics and outcomes of the 85 eligible studies divided into static stretching and foam rolling studies.

**Table 1:** Study characteristics

Study	Mean age (years)	Sex	Intervention duration in weeks	Intervention frequency per week	Intervention duration per bout (s)	Outcome
*Static stretching studies*						
Aquino et al. (2010) [33]	22.5	Mi	8	3	120	Passive KE
Ayala et al (2013) [34]	22.1	M	12	3	180	Passive SLR
Ayala et al. (2010) [35]	21.3	Mi	12	3	180	Lower extremity following 4–8–12 weeks
Bandy et al. (1998) [36]	26.7	NR	6	5	30	Passive KE
Bandy et al. (1997) [37]	26.56	Mi	6	5	30, 60, 90, and 180	Passive KE
Barbosa et al. (2018) [38]	21.77	M	4	3	180	Active SLR
Batista et al. (2009) [39]	67.6	F	4	2	420	Passive KE
Ben and Harvey (2010) [40]	37	Mi	6	5	1800	Passive HF
Blazevich et al. (2014) [41]	18.6	M	3	7	240	DF
Bybee et al. (2008) [42]	30	N	8	7	240	Lumbar extension
Chan et al. (2001) [43]	20	Mi	4 and 8	3	150 and 300	Passive KE
Chepeha et al. (2018) [44]	20.3	Mi	8	7	600	Shoulder IR and horizontal adduction
Cipriani et al. (2012) [45]	NR	Mi	4	3, 6, 7, and 14	60	Static HF
Covert et al. (2010) [46]	21.8	Mi	4	3	30	KE
da Costa et al. (2013) [47]	67.5	Mi	12	2	240	HF, HE, and DF
de Baranda et al. (2010) [48]	21	Mi	12	3	180	SLR
de Castro et al. (2013) [49]	13.3	M	12	3	90	Hip IR and ER
Donti et al. (2021) [50]	9.6	F	9	3	180	HE
Feland et al. (2001) [51]	84.9	Mi	6	5	60, 120, and 240	Passive KE
Feland et al. (2010) [52]	23.5	Mi	4	5	300	Passive KE
Gajdosik et al. (2005) [53]	74.2	F	8	3	150	DF
Gajdosik et al. (2007) [54]	22	F	6	5	300	Passive DF
Godges et al. (1993) [55]	21	M	3	2	360	Thomas Test
González-Ravé et al. (2012) [56]	65.8	Mi	13	2	27 and 30	Passive shoulder flexion and HF
Gribble et al. (1999) [57]	19.67	Mi	6	3	120	Passive HF
Gunaydin et al. (2020) [58]	22.9	Mi	6	3	NR	Passive KE
Hadjicharalambous (2016) [59]	16.05	M	4	4	190	SR
Ikeda et al. (2019) [60]	22	M	6	3	180	KF
Johnson et al. (2014) [61]	22	Mi	6	6	90	Passive KE
Kerrigan et al. (2003) [62]	65	M	10	14	240	HE and PF
Knapik et al. (2019) [63]	15.95	Mi	12	3	600	Passive DF
Kokkonen et al. (2007) [64]	23	Mi	10	3	45	SR
Konrad and Tilp (2014) [25]	22.9	Mi	6	5	120	Passive DF
Li et al. (1996) [65]	28.9	Mi	3	7	150	SLR and active KE
Lobel (2016) [66]	25.47	F	3	4	32 and 240	Modified Thomas Test
Longo et al. (2021) [67]	22.7	Mi	12	5	450	Passive DF
Gallo et al. (2013) [68]	66.4	F	16	3	180 and 360	SR
Maciel and Câmara (2008) [69]	22.14	F	2	5	90	Passive KE
Marshall et al. (2011) [70]	22.7	Mi	4	5	900	SLR
Mahieu et al. (2007) [71]	22.1	Mi	6	7	100	DF
Mayorga-Vega et al. (2014) [72]	10.3	Mi	8	2	360	SR
Mayorga-Vega et al. (2014) [73]	9.89	Mi	8	2	240	SR
Mayorga-Vega et al. (2015) [74]	12.6	Mi	8	1	240	SR
Mayorga-Vega et al. (2016) [75]	8.45	Mi	9	2	240	Hams flexibility
Mayorga-Vega et al. (2017) [76]	9	Mi	34	1	180	Hams flexibility
McClure et al. (2007) [77]	23.2	Mi	4	7	150	Shoulder IR and ER
Melo et al. (2021) [78]	23.7	M	4	3	90	Passive and active KE
Merino-Marban et al. (2015) [79]	5.9	Mi	8	2	20	SR
Morton et al. (2011) [80]	21.92	Mi	5	NR	NR	KE, HF, HE, and shoulder extension
Muyor et al. (2012) [81]	44.2	F	12	3	20	Active HF and passive SLR
Nakamura et al. (2017) [82]	23.8	M	4	3	120	Passive DF
Nakamura et al. (2021) [6]	21.65	M	4	3	180	Passive DF
Oba et al. (2021) [83]	22.9	M	5	1	400	Passive DF
Panidi et al. (2021) [3]	13.5	F	12	5	900	DF
Piqueras-Rodriguez et al. (2016) [84]	12.3	M	8	1	540	Passive Hams flexibility
Reid and McNair (2004) [85]	15.8	M	6	5	90	Passive KE
Reiner et al. (2023) [86]	27.4	Mi	7	3	900	Active shoulder extension
Roberts and Wilson (1999) [87]	20.5	Mi	5	3	45	Active and passive HF, KF, and KE
Rodríguez et al. (2008) [88]	11.8	Mi	32	2	NR	SR
Santonja Medina et al. (2007) [89]	10.5	Mi	31	2 and 4	300	Passive SLR
Sermaxhaj et al. (2021) [90]	13.9	NR	17	3	340	Static SR
Simão et al. (2011) [91]	34	F	16	3	NR	SR
Stanziano et al. (2009) [92]	88.8	Mi	8	2	90,135, and 180	Back scratch test, SR, KE, and total body rotation
Warneke et al. (2022) [93]	27	Mi	6	7	3600	Knee-to-wall, passive DF
Warneke et al. (2023) [94]	27.4	Mi	6	7	3600	Knee-to-wall, passive DF
Warneke et al. (2023) [95]	25.9	Mi	6	7	600,1800, and 3600	Knee-to-wall, passive DF
Webright et al. (1997) [96]	21.6	Mi	6	14	30	Active KE
Wohlann et al. (2023) [97]	25.4	Mi	6	7	300, 600, and 300	Knee-to-wall test, SLR, modified Thomas Test
Yildirim et al. (2016) [98]	21.5	Mi	4	3	300	HF
Youdas et al. (2003) [99]	36.4	Mi	6	7	30, 60, and 120	Active DF
Yuktasir and Kaya (2009) [100]	21.82	M	6	4	120	KE
Zaidi et al. (2023) [101]	58.8	M	4	3	80	Active knee ROM, SR
*Foam rolling studies*						
Boguszewski et al. [102]	23.6	F	8	2	nr	SR
Guillott et al. [103]	18.85	M	7	~ 2,1	20 or 40	Side split, active straight leg, active flexed leg raising of the hip, active HE, active KE, active dorsiflexion
Hodgson et al. [104]	25	Mi	4	3	120	Hamstrings active ROM, Hamstrings passive ROM, Quadriceps active ROM, Quadriceps passive ROM
Junker and Stöggl [23]	30.5	M	4	3	105	Stand and reach
Junker and Stöggl [105]	29.8	Mi	8	2	95	SR
Kiyono et al. [27]	20.8	Mi	5	3	90	Dorsiflexion ROM
Le Gal et al. [106]	15	Mi	5	3	180	Glenohumeral internal ROM
Li et al. [21]	37.25	Mi	8	1,4	nr	KF ROM
Miller and Rockey [107]	20.53	Mi	8	3	180	Active KE
Sandrey et al. [108]	21.1	Mi	3	2	120	KF and KE ROM
Seever et al. (2022) [109]	24.3	Mi	2	6	60	Active DF (weight-bearing lunge test)
Shalamzari et al. (2022) [110]	24.9	Mi	8	3	26	Knee ROM
Stovern et al. [111]	20.8	Mi	6	3	60	Dorsiflexion and KF ROM, SR

*A/R* active or recreational, *DF* dorsiflexion, *E/P* elite or professional, *ER* external rotation, *F* female, *FS* fully supervised, *GM* gluteus maximus, *GN* gastrocnemius, *HA* hip adductors, *Hab* hip abductors, *Hams* hamstrings, *HE* hip extension, *HF* hip flexion, *IR* internal rotation, *KE* knee extension, *KF* knee flexion, *LD* latissimus dorsi, *M* male, *Mi* mixed, *N/Mi* not stated or mixed, *NR* not reported, *NS* not supervised, *PF* plantar flexion, *PS* partially supervised, *Quads* quadriceps, *ROM* range of motion, *SLR* straight-leg raise, *SOL* soleus, *SR* sit-and-reach, *S/U* stationary or untrained, *TFL* tensor facia late, *TS* triceps surae

### Risk of Bias Assessment and Methodological Quality

**Fig. 2:**
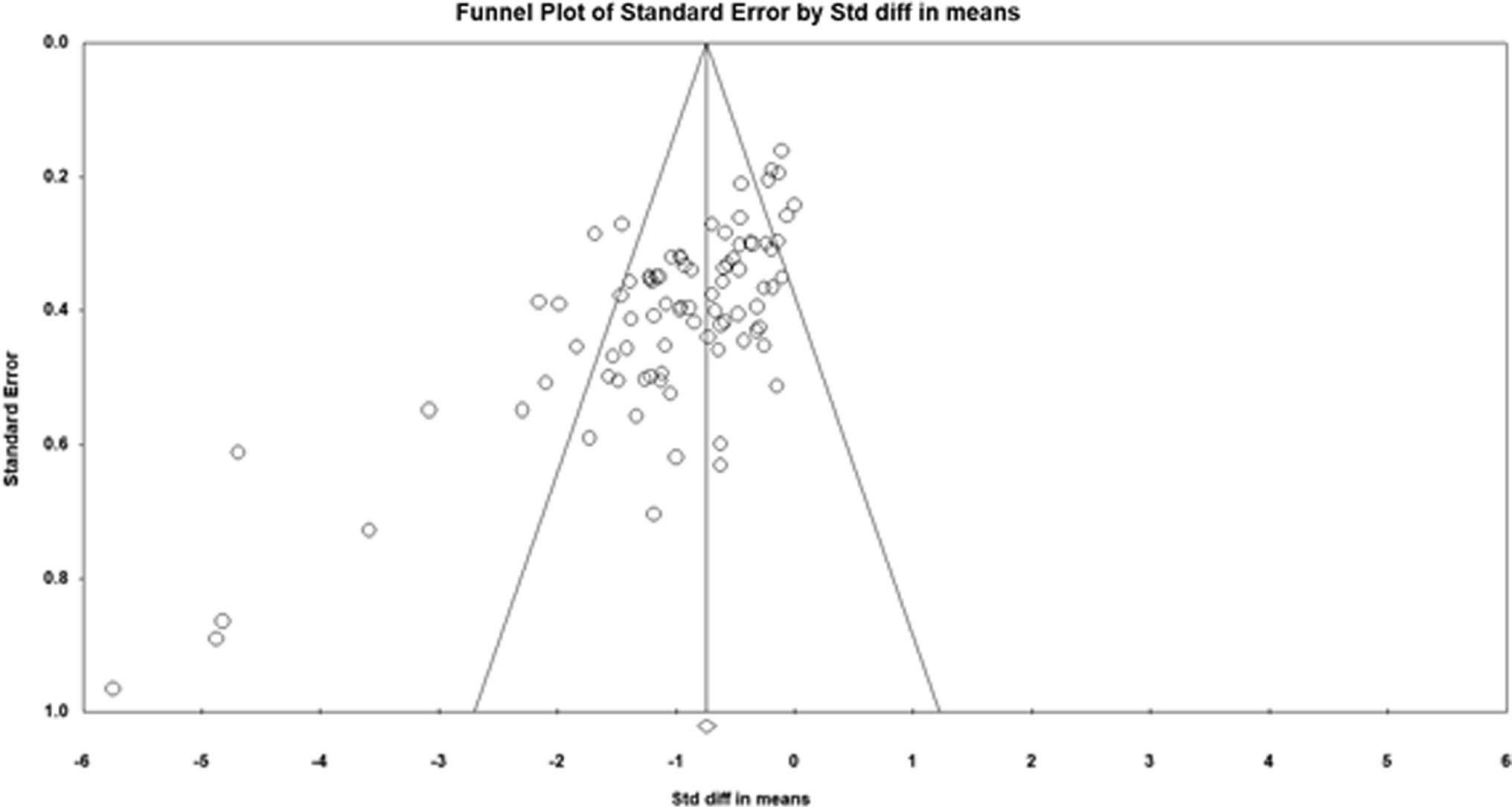
Funnel plot analysis of all included studies on foam rolling and static stretching. *Std diff* standard difference

Figure 2 shows the funnel plot, including all 85 studies in this systematic review and meta-analysis. A visual inspection of the funnel plot and the Egger’s regression intercept test (intercept − 4.139; *p* < 0.001) indicated reporting bias. The methodological quality, as assessed by the PEDro scale, revealed a range of scores between 4 and 10 points (out of 11) for all the included studies. The average PEDro scale score value was 7.3 (± 1.1), indicating a low risk of bias [20]. The assessors agreed with 99.1% out of the 935 criteria (85 studies × 11 scores). The mismatched outcomes were discussed, and the assessors agreed on the scores presented in Table 2.

**Table 2:** PEDro scale results

Study	1	2	3	4	5	6	7	8	9	10	11	Total
*Static stretching studies*												
Aquino et al. (2010)[33]	Y	Y	N	Y	N	N	Y	Y	Y	Y	Y	8
Ayala et al. (2010) [35]	Y	Y	N	Y	N	N	N	Y	Y	Y	Y	7
Ayala et al. (2013) [34]	Y	N	N	N	N	Y	Y	Y	Y	Y	Y	7
Bandy et al. (1998) [36]	Y	Y	N	Y	N	N	N	Y	Y	Y	Y	7
Bandy et al. (1997) [37]	Y	Y	N	Y	N	N	N	Y	Y	Y	Y	7
Barbosa et al. (2018) [38]	Y	Y	N	Y	Y	N	N	N	Y	Y	Y	7
Batista et al. (2009) [39]	Y	N	N	Y	N	N	N	N	Y	Y	Y	5
Ben and Harvey (2010) [40]	Y	Y	Y	Y	N	Y	Y	Y	Y	Y	Y	10
Blazevich et al. (2014) [41]	Y	Y	N	Y	N	N	N	Y	Y	Y	Y	7
Bybee et al. (2008) [42]	Y	Y	N	Y	Y	N	N	Y	Y	Y	Y	8
Chan et al. (2001) [43]	Y	Y	N	Y	N	N	N	Y	Y	Y	Y	7
Chepeha et al. (2018) [44]	Y	Y	N	Y	N	N	Y	Y	Y	Y	Y	8
Cipriani et al. (2012) [45]	Y	Y	N	Y	N	Y	Y	Y	Y	Y	Y	9
Covert et al. (2010) [46]	Y	Y	Y	Y	N	N	Y	Y	Y	Y	Y	9
Da Costa et al. (2013) [47]	Y	N	N	Y	N	N	N	N	Y	Y	Y	5
de Baranda et al. (2010)[48]	Y	Y	Y	Y	Y	N	Y	Y	Y	Y	N	9
De Castro et al., 2013 [49]	Y	Y	N	Y	N	N	N	Y	Y	Y	Y	7
Donti et al. (2021) [50]	N	Y	N	Y	N	N	N	Y	Y	Y	Y	6
Feland et al. (2001) [51]	Y	Y	N	Y	N	N	N	Y	Y	Y	Y	7
Feland et al. (2010) [52]	Y	Y	N	Y	N	N	Y	Y	Y	Y	Y	8
Gajdosik et al. (2005) [53]	Y	Y	N	Y	N	N	Y	Y	Y	Y	Y	8
Gajdosik et al. (2007) [54]	N	Y	N	Y	N	N	Y	Y	Y	Y	Y	7
Godges et al. (1993) [55]	Y	Y	N	Y	N	N	N	Y	Y	Y	Y	7
Gonzalez-Rave et al. (2012) [56]	Y	Y	N	Y	N	N	N	Y	Y	Y	Y	7
Gribble et al. (1999) [57]	Y	Y	N	Y	N	N	N	Y	Y	Y	Y	7
Gunaydin et al. (2020) [58]	Y	Y	N	Y	N	N	N	Y	Y	Y	Y	7
Hadjicharalambous (2016) [59]	Y	Y	N	Y	N	N	N	Y	Y	Y	Y	7
Ikeda et al. (2019) [60]	Y	Y	N	Y	N	N	N	Y	Y	Y	Y	7
Johnson et al. (2014) [61]	Y	Y	N	Y	N	N	N	Y	Y	Y	Y	7
Kerrigan et al. (2003) [62]	Y	Y	Y	Y	N	Y	Y	Y	Y	Y	Y	10
Knapik et al. (2019) [63]	Y	Y	N	Y	N	N	N	Y	Y	Y	Y	7
Kokkonen et al. (2007) [64]	Y	Y	Y	Y	N	N	N	Y	Y	Y	Y	8
Konrad and Tilp (2014) [25]	Y	Y	N	Y	N	N	Y	Y	Y	Y	Y	8
Li et al. (1996) [65]	Y	Y	N	Y	N	N	N	Y	Y	Y	Y	7
Lobel (2016) [66]	Y	Y	N	Y	N	N	N	Y	Y	Y	Y	7
Longo et al. (2021) [67]	Y	Y	N	Y	N	N	N	Y	Y	Y	Y	7
Gallo et al. (2013) [68]	Y	N	N	Y	N	N	N	Y	Y	Y	Y	6
Maciel and Câmara (2008) [69]	Y	Y	N	Y	Y	N	N	Y	Y	Y	Y	8
Marshall et al. (2011) [70]	Y	Y	N	N	N	N	N	Y	Y	Y	Y	6
Mahieu et al. (2007) [71]	Y	Y	N	Y	N	N	Y	Y	Y	Y	Y	8
Mayorga-Vega et al. (2014) [72]	Y	Y	N	Y	N	N	N	Y	Y	Y	Y	7
Mayorga-Vega et al. (2014) [73]	Y	Y	N	Y	N	N	N	Y	Y	Y	Y	7
Mayorga-Vega et al. (2015) [74]	Y	Y	N	Y	N	N	N	Y	Y	Y	Y	7
Mayorga-Vega et al. (2016) [75]	Y	Y	N	Y	N	N	N	Y	Y	Y	Y	7
Mayorga-Vega et al. (2017) [76]	Y	Y	N	Y	N	N	N	Y	Y	Y	Y	7
McClure et al. (2007) [77]	Y	Y	N	Y	N	N	Y	Y	Y	Y	Y	8
Melo et al. (2021) [78]	Y	Y	Y	Y	N	N	Y	Y	Y	Y	Y	9
Merino-Marban et al. (2015) [79]	Y	Y	N	Y	N	N	N	Y	Y	Y	Y	7
Morton et al. (2011) [80]	Y	Y	N	Y	N	N	N	Y	Y	Y	Y	7
Muyor et al. (2012) [81]	Y	Y	N	Y	N	N	N	Y	Y	Y	Y	7
Nakamura et al. (2017) [82]	Y	Y	N	Y	N	Y	Y	Y	Y	Y	Y	9
Nakamura et al. (2021) [6]	Y	Y	N	Y	N	N	N	Y	Y	Y	Y	7
Oba et al. (2021) [83]	Y	Y	N	Y	N	N	N	Y	Y	Y	Y	7
Panidi et al. (2021) [3]	Y	Y	N	Y	N	Y	Y	Y	Y	Y	Y	9
Piqueras-Rodriguez et al. (2016) [84]	Y	Y	N	Y	N	N	Y	Y	Y	Y	Y	8
Reid and McNair (2004) [85]	Y	Y	N	Y	N	Y	Y	Y	Y	Y	Y	9
Reiner et al. (2023) [86]	Y	Y	Y	Y	N	N	N	N	Y	Y	Y	7
Roberts and Wilson (1999) [87]	N	Y	N	Y	N	N	Y	Y	Y	Y	Y	7
Rodriguez et al. (2008) [88]	Y	Y	N	Y	N	N	N	Y	Y	Y	Y	7
Santonja Medina et al. (2007) [89]	Y	Y	N	Y	Y	Y	Y	Y	Y	Y	Y	10
Sermaxhaj et al. (2021) [90]	N	N	N	N	N	N	N	Y	Y	Y	Y	4
Simão et al. (2011) [91]	Y	Y	N	Y	N	N	N	Y	Y	Y	Y	7
Stanziano et al. (2009) [92]	Y	Y	N	Y	N	N	N	Y	Y	Y	Y	7
Warneke et al. (2022) [93]	Y	Y	N	Y	N	N	N	Y	Y	Y	Y	7
Warneke et al. (2023) [94]	Y	Y	N	Y	N	N	N	Y	Y	Y	Y	7
Warneke et al. (2023) [95]	Y	Y	N	Y	N	N	N	Y	Y	Y	Y	7
Webright et al. (1997) [96]	Y	Y	N	Y	N	N	N	Y	Y	Y	Y	7
Wohlann et al. (2023) [97]	Y	N	N	Y	N	N	Y	Y	Y	Y	Y	7
Yildirim et al. (2016) [98]	Y	Y	N	Y	N	Y	N	Y	Y	Y	Y	8
Youdas et al. (2003) [99]	Y	Y	N	Y	N	N	Y	Y	Y	Y	Y	8
Yuktasir and Kaya (2009) [100]	Y	Y	N	Y	N	Y	Y	Y	Y	Y	Y	9
Zaidi et al. (2023) [101]	Y	Y	N	Y	Y	N	N	Y	Y	Y	Y	8
*Foam rolling studies*												
Boguszewski et al. [102]	Y	Y	N	Y	N	N	N	Y	Y	Y	Y	6
Guillott et al. [103]	Y	Y	N	Y	Y	N	Y	Y	Y	Y	Y	8
Hodgson et al. [104]	Y	Y	N	Y	N	N	N	Y	Y	Y	Y	6
Junker and Stöggl [23]	Y	Y	N	Y	N	N	N	Y	Y	Y	Y	6
Junker and Stöggl [105]	Y	Y	N	Y	N	N	N	Y	Y	Y	Y	6
Kiyono et al. [27]	Y	Y	N	Y	N	N	N	Y	Y	Y	Y	6
Le Gal et al. [106]	Y	Y	N	Y	N	N	Y	Y	Y	Y	Y	7
Li et al. [21]	Y	Y	Y	Y	N	N	Y	Y	Y	Y	Y	8
Miller and Rockey [107]	Y	Y	N	Y	N	N	N	Y	Y	Y	Y	6
Sandrey et al. [108]	Y	Y	N	Y	N	N	N	Y	Y	Y	Y	6
Seever et al. (2022) [109]	Y	Y	Y	Y	N	Y	N	Y	Y	Y	Y	9
Shalamzari et al. (2022) [110]	Y	Y	N	Y	Y	N	Y	Y	Y	Y	Y	9
Stovern et al. [111]	Y	N	N	Y	N	N	N	Y	Y	Y	Y	5

PEDro scale score criteria. (1) Eligibility criteria were specifed. (2) Subjects were randomly allocated to groups (in a crossover study, subjects were randomly allocated an order in which treatments were received). (3) Allocation was concealed. (4) The groups were similar at baseline regarding the most important prognostic indicators. (5) There was blinding of all subjects. (6) There was blinding of all therapists/researchers who administered the therapy/protocol. (7) There was blinding of all assessors who measured at least one key outcome. (8) Measures of at least one key outcome were obtained from more than 85% of the subjects initially allocated to groups. (9) All subjects for whom outcome measures were available received the treatment or control condition as allocated or, where this was not the case, data for at least one key outcome were analyzed by “intention to treat.” (10) The results of between-group statistical comparisons were reported for at least one key outcome. (11) The study provided both point measures and measures of variability for at least one key outcome

*N* no, *Y* yes

### Main Analysis

**Fig. 3:**
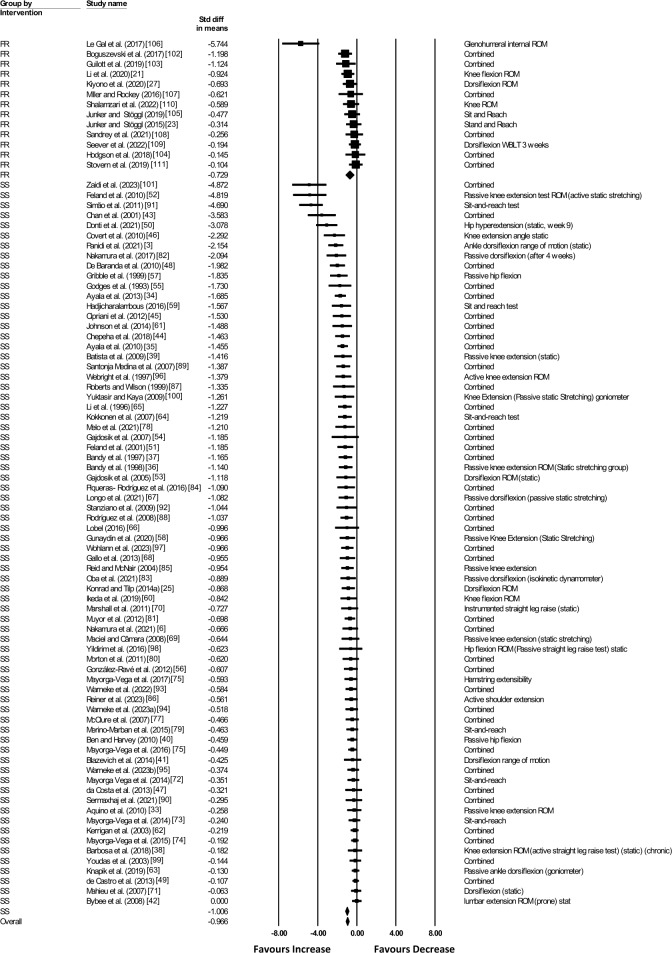
Forest plot presenting all included studies investigating either the effects of foam rolling (FR) or static stretching (SS) on range of motion (ROM). *CI* confidence interval, *combined* mean of the selected outcomes of one study, *Std diff in means* standardized difference in means, *WBLT* weight-bearing lunge test

The main meta-analysis showed a significant moderate ES (ES =  − 1.006; *Z* =  − 11.544; 95% confidence interval [CI] − 1.177 to − 0.835; *p* < 0.001; *I*^2^ = 76.193) increase in joint ROM following static stretching only compared with a control condition. Similarly, there was a significant moderate ES (ES =  − 0.729; *Z* =  − 3.435; 95% CI − 1.145 to − 0.313; *p* = 0.001; *I*^2^ = 69.206) increase in joint ROM following foam rolling only compared with a control condition (see Fig. 3). The comparison between the ESs of static stretching and foam rolling revealed no statistically significant difference according to the *Q*-statistics (*Q* = 1.453; *df* (Q) = 1; *p* = 0.228).

### Moderating Variables

#### Weeks of Intervention (> 4 Weeks and ≤ 4 Weeks)

Considering studies with an overall duration of ≤ 4 weeks, 19 studies on static stretching and four studies on foam rolling were included in the analysis. While static stretching compared with controls showed a significant increase in joint ROM (ES =  − 1.436; *Z* =  − 6.371; 95% CI − 1.877 to − 0.994; *p* < 0.001; *I*^2^ = 74.997), this was not evident for foam rolling (ES =  − 0.229; *Z* =  − 1.155; 95% CI − 0.616 to 0.159; *p* = 0.248; *I*^2^ = 0.000). The comparison between the ESs of static stretching and foam rolling revealed a statistically significant difference according to the *Q*-statistics (*Q* = 16.203; *df* (*Q*) = 1; *p* < 0.001), indicating a greater effect for increasing the ROM with less than 4 weeks of static stretching training.

With regard to studies with an overall duration of > 4 weeks, 54 studies on static stretching and nine studies on foam rolling were included in the analysis. Both static stretching (ES =  − 0.919; *Z* =  − 9.807; 95% CI − 1.103 to − 0.735; *p* < 0.001; *I*^2^ = 76.436) as well as foam rolling (ES =  − 1.007; *Z* =  − 3.415; 95% CI − 1.585 to − 0.429; *p* = 0.001; *I*^2^ = 75.767) showed an increase in ROM compared with a control condition. The comparison between the EFs of static stretching and foam rolling revealed no statistically significant difference according to the *Q*-statistics (*Q* = 0.081; df (*Q*) = 1; *p* = 0.776).

#### Sex

As only two studies on foam rolling included female participants, no comparison was done between the ESs of static stretching and foam rolling on joint ROM. Considering male participants, 16 studies on static stretching and four studies on foam rolling were identified. Male participants showed an increase in ROM following both static stretching (ES =  − 1.012; *Z* =  − 5.072; 95% CI − 1.403 to − 0.621; *p* < 0.001; I^2^ = 79.579) and foam rolling (ES =  − 0.648; *Z* =  − 2.755; 95% CI − 1.109 to − 0.187; *p* = 0.006; I^2^ = 0.000) compared with a control condition. The comparison between the ESs of static stretching and foam rolling revealed no statistically significant difference according to the *Q*-statistics (*Q* = 1.395; *df* (*Q*) = 1; *p* = 0.238).

#### Total Intervention Duration

As only two studies on foam rolling with a ≥ 3600-s intervention duration were identified, no comparison was performed between the ESs of static stretching and foam rolling on joint ROM with this intervention duration. Considering studies with a < 3600-s intervention duration, 33 studies on static stretching and nine studies on foam rolling were included in the analyses. Studies with a < 3600-s intervention duration showed an increase in ROM following static stretching (ES =  − 1.013; *Z* =  − 8.514; 95% CI − 1.246 to − 0.780; *p* < 0.001; *I*^2^ = 68.591) as well as following foam rolling (ES =  − 0.719; *Z* =  − 2.401; 95% CI − 1.306 to − 0.132; *p* = 0.016; *I*^2^ = 76.842) compared with a control condition. The comparison between the ESs of static stretching and foam rolling revealed no statistically significant difference according to the *Q*-statistics (*Q* = 0.830; *df* (*Q*) = 1; *p* = 0.362).

## Discussion

The main aim of this systematic review and meta-analysis was to compare the long-term effects of static stretching with foam rolling on joint ROM. When including all eligible studies in the meta-analysis, no significant ROM difference (*p* = 0.228) in the ESs between static stretching (ES =  − 1.006) and foam rolling (ES =  − 0.729) was detected. Additionally, if only studies with a low volume (< 3600-s intervention duration) or only male participants were considered, no significant difference in the increase in joint ROM was detected between the two methods. However, static stretching was more effective in increasing ROM compared with foam rolling when a study duration ≤ 4 weeks was applied (*p* < 0.001).

In a previous review, a direct comparison of the long-term effects of stretching and foam rolling on ROM was performed with three ESs only [9], raising questions about the robustness of this result. This three-study meta-analysis showed no significant difference between stretching and foam rolling on ROM (ES = 0.516; *p* = 0.12). Additionally, it has to be noted that out of those three studies, two studies applied static stretching [21, 22] while one applied proprioceptive neuromuscular facilitation stretching [23]. Although there was no direct comparisons between static stretching and foam rolling within the eligible studies of a current review as it was in Konrad et al. [9], the comparison of the ESs of static stretching and foam rolling in the meta-analysis showed as well no significant difference between the modalities (*p* = 0.228). However, it should be noted that there was still a slight discrepancy between the ESs of static stretching (ES =  − 1.006) and foam rolling (ES =  − 0.729). A potential explanation for the slightly favorable effects of static stretching compared with foam rolling as seen in the meta-analysis by Konrad et al. [9] and in the findings of the current meta-analysis might be that with static stretching the whole muscle–tendon unit is under tension throughout the stretch, while during foam rolling only the rolled area of the muscle–tendon unit is under tension. Consequently, it can be assumed that the muscle–tendon unit receives more consistent loading or (longitudinal) tension with static stretching compared with foam rolling.

A slightly higher ES but still nonsignificant difference in static stretching (ES =  − 1.013) compared with foam rolling (ES =  − 0.719) on ROM was shown, when only studies with a lower volume (< 3600 s) were compared. It has to be noted that the studies on foam rolling mainly used a low-volume approach. The total intervention volume in the eligible foam rolling studies was in a range between 300 and 4320 s, while for the eligible static stretching studies the range was 320 s to 151,200 s. Total volume can be indeed a crucial variable as studies on high-volume SS training (e.g., ≥ 30 min stretching a week) reported changes in the muscle–tendon unit structure [2, 3, 24]. Such changes have not been seen in lower volume static stretching studies with, for example, 10 min stretching per week [25] as well as in the previous foam rolling studies. More precisely, a recent meta-analysis reported no changes in muscle performance following long-term lower volume foam rolling training [26]. The effect mechanism for the ROM increase following foam rolling has been suggested to be related to stretch tolerance rather than changes in muscle stiffness [27, 28]. Possibly, a higher volume foam rolling approach might lead to further changes in muscle–tendon function (e.g., muscle strength) as well as changes in muscle–tendon structure (e.g., decrease in stiffness). Consequently, future studies on foam rolling should take this into account and perform a foam rolling protocol with a much higher volume than applied in previous studies (e.g., > 3600 s).

According to a further moderator analysis, the weeks of intervention seemed to be a very crucial factor in increases in ROM. While there was no significant difference between static stretching and foam rolling training on the effects of joint ROM when the duration of the studies was more than 4 weeks, a significantly favorable effect for static stretching compared with foam rolling was shown with a total intervention duration ≤ 4 weeks. However, there was no significant increase in joint ROM following foam rolling within the first 4 weeks of the intervention. This might be explained by the finding that the mean total intervention duration for the 19 studies with SS was 3420 s while for the four foam rolling training studies it was only 1692 s. Consequently, time under tension seems to be an important factor as well. However, by just taking the current evidence into account, it can be suggested to use static stretching rather than foam rolling if the goal is to increase the ROM within the first 4 weeks.

However, it has to be mentioned at this point that techniques other than static stretching or foam rolling can increase the ROM of a joint in the long term. Alizadeh et al. [8] showed in their meta-analysis that frequent resistance training performed within the full ROM can increase joint ROM long term. In addition to the increase in ROM, resistance training has other beneficial effects such as increases in muscle strength and mass, reducing back pain, and enhancing cardiovascular health [29].

Finally, the last moderator analysis (i.e., sex) indicated no significant difference between the ESs of static stretching and foam rolling training within male participants. However, it has to be noted that no such comparison could be performed with female participants. Consequently, to overcome such a sex research gap [30], future studies should either report sex-specific results or conduct studies with female participants only.

The funnel plot as well as the Egger’s regression intercept test (intercept − 4.139; *p* < 0.001) indicated a reporting bias limitation. It is clearly established that significant “positive” results are more likely to be published with an increased probability that they would be published in higher impact journals and thus also achieve a higher number of citations [31, 32]. Although one must always be cautious when interpreting results, especially those with a possibility of bias, all the significant ESs in the current systematic review and meta-analysis showed a moderate ES.

## Conclusions

The main analysis with all the eligible studies revealed that both static stretching as well as foam rolling can increase joint ROM with a moderate magnitude. Although the ES in static stretching for increasing the ROM is slightly higher compared with foam rolling, this difference was not significant. Considering only studies with an intervention duration ≤ 4 weeks, foam rolling was ineffective for increasing joint ROM and hence, static stretching was shown to be more effective. Other moderators such as sex as well as the total intervention duration showed no significant difference between the two modalities. According to the results, it can be recommended to use SS training if the training duration is scheduled for ≤ 4 weeks. Future studies should explore the effects of high-volume foam rolling training, foam rolling training in exclusively female participants, as well as SS training and foam rolling training on the upper limbs.

## Declarations

### Availability of data and material

All data will be made available on request to the corresponding author.
